# Prevalence of adolescent physical activity-related injuries in sports, leisure time, and school: the National Physical Activity Behaviour Study for children and Adolescents

**DOI:** 10.1186/s12891-018-1969-y

**Published:** 2018-02-15

**Authors:** Anu M. Räisänen, Sami Kokko, Kati Pasanen, Mari Leppänen, Arja Rimpelä, Jari Villberg, Jari Parkkari

**Affiliations:** 1 Tampere Research Center of Sports Medicine, UKK Institute for Health Promotion Research, P.O. BOX 30, 33501 Tampere, Finland; 2 Research Center for Health Promotion, Faculty of Sport and Health Sciences, University of Jyväskylä, Jyväskylä, Finland; 3 Sport Injury Prevention Research Centre, Faculty of Kinesiology, University of Calgary, Calgary, AB Canada; 4 Faculty of Social Sciences, Health Sciences and PERLA (Tampere Centre for Childhood, Youth and Family Research), University of Tampere, Tampere, Finland; 5 Department of Adolescent Psychiatry, Tampere University Hospital, Tampere, Finland

**Keywords:** Athletic injury, Injury prevention, Youth, Adolescents, Safety, Physical activity

## Abstract

**Background:**

The purpose of this study was to investigate the prevalence of adolescent physical activity-related injuries in sports club activities, leisure time physical activity and school-based physical activity. The secondary aim was to investigate the differences in the prevalence of physical activity -related injuries between years 2014 and 2016. In addition, we set out to study the associations between age, sex and the frequency of physical activity and injury prevalence.

**Methods:**

This cross-sectional study is based on the National Physical Activity Behaviour Study for Children and Adolescents (LIITU in Finnish) conducted in years 2014 and 2016. The subjects completed an online questionnaire in the classroom during school hours. A total of 8406 subjects participated in the current study. Out of these, 49% were boys and 51% were girls. The proportions of 11-, 13-, and 15-year-olds were 35%, 34% and 31%, respectively.

**Results:**

In the combined data for 2014 and 2016, injury prevalence was higher in sports club activities (46%, 95% CI 44.8–47.8) than in leisure time PA (30%, 95% CI, 28.5–30.5) or school-based PA (18%, 95% CI, 17.4–19.1). In leisure time PA, the injury prevalence was higher than in school-based PA. In all the three settings, injury prevalence was higher in 2016 than in 2014. Frequency of PA was associated with a higher risk for PA-related injuries in sports clubs and leisure time.

**Conclusions:**

With half of the subjects reporting at least one PA-related injury during the past year, results indicate that adolescent PA-related injuries are a large-scale problem. There is a worrisome rise in injury prevalence in recent years. From a public health standpoint, there is an urgent need to invest in injury prevention to reverse this trend.

## Background

With the high levels of physical inactivity around the world, increasing physical activity (PA) has become a key element of public health promotion [1]. Physical activity provides a great deal of benefits to health and well-being [2–4]. So great in fact that it is promoted as a medicine or a drug against life-style related diseases [5]. Nonetheless, PA as health promotion tool is not one without adverse effects. Participation in PA is the main cause of adolescent unintentional injuries in many developed countries [6–9]. Participating in organized sports in adolescence is a major risk factor for hospitalization throughout adolescence and on to early adulthood [10]. However, most injuries can be prevented.

A recent study by Richmond et al. [11] demonstrated that a school-based neuromuscular training intervention reduces the risk of PA-related injuries while simultaneously improving health markers. Reducing the rates of leisure time PA injuries among adolescents is also possible, as has been proven by bicycle helmet use [12]. Furthermore, several studies have shown that it is possible to prevent adolescent injuries in organized sports by neuromuscular training [13–17].

With most studies focusing on injuries in organized sports, there is still limited knowledge on PA-related injuries in other settings. Some previous studies have investigated the injuries in school children in sports, leisure time and school sports [18, 19]. In our recent study [20] on adolescent injuries we observed the highest injury prevalence in sports club activities, followed by leisure time PA and school-based PA.

One established risk factor for PA-related injury is a previous PA-related injury [21–23]. Therefore, one of the most efficient ways to reduce injury rates is to prevent the first injuries. Injuries can have short and long term consequences on adolescent health. In short term, an injury can lead to unfavorable weight gain and negative changes on obesity markers [24, 25]. In long term, the risk of osteoarthrosis later in life is increased [26]. The fear of further injury can limit participation and lead to dropping out of PA. Because of reduced activity, individuals will eventually lose the health benefits produced by PA and this will have negative effects on public health. To achieve best possible results, all PA promotion efforts should include injury prevention in to their agenda [27, 28].

The purpose of this study was to investigate the prevalence of adolescent PA-related injuries in sports club activities, leisure time PA and school-based PA. The secondary aim of the study was to investigate the differences in the prevalence of PA-related injuries between 2014 and 2016. In addition, we set out to study the associations between age, sex and the frequency of PA and injury prevalence.

## Methods

This cross-sectional investigation is based on the National Physical Activity Behaviour Study for Children and Adolescents (LIITU in Finnish) conducted in years 2014 and 2016. The first data collection in 2014 was carried out in connection with the World Health Organization Cross-national Health Behaviours in School-Aged Children study (HBSC). In 2016, the National Physical Activity Behaviour Study for Children and Adolescents was carried out independently. For both data collections, a random sample of schools was drawn from the Statistics Finland database based on the HBSC protocol [29]. The subjects completed the online questionnaire in the classroom during a lesson. The questionnaire included questions on several aspects of PA participation. The current study is based on the questions on PA-related injuries.

In 2014, 195 schools (3452 subjects) agreed to participate in the study. A total of 3071 subjects replied to the online questionnaire, making the response rate 89%. Due to incomplete or inconsistent answers, 269 subjects were excluded and 2802 subjects were included in the study. In 2016, 285 Finnish-language schools (10,513 subjects) and 65 Swedish-language schools (1975 subjects) agreed to participate and 7565 subjects replied to the online questionnaire, making the response rate 61%. Out of these, 174 were excluded due to incomplete or inconsistent answers. In addition, the youngest subjects (9 years, *n* = 1787) did not participate in the injury part of the questionnaire and were excluded from the current study. In 2016, a total of 5604 subjects were included in the study.

A total of 8406 subjects were included in the current study. Out of these, 49% were boys and 51% were girls. The proportions of 11- (5th graders), 13- (7th graders) and 15-year-olds (9th graders) were 34.6%, 34.4% and 31.0%, respectively.

### Physical activity-related injuries

The injury prevalence refers to the proportion of subjects who were injured in physical activities during the past twelve months. No definition for injury was provided in the questionnaire. The injury prevalence in three settings were based on three questions: ‘During the past year, have you suffered an injury in sports club activities?’, ‘During the past year, have you suffered an injury in leisure time physical activities (not in a sports club)?’ and ‘During the past year, have you suffered an injury in a physical education class or instructed student sports?’. Four options were provided: ‘No’, ‘Once’, ‘Twice’, and ‘Three times or more’. The question about sports club injuries was only asked from the subjects who had reported participating in sports club activities in an earlier question (54% in 2014 and 62% in 2016). Questions regarding leisure time PA and school-based PA injuries were asked from all subjects.

The injury prevalence in sports club activities was calculated as a percentage of subjects reporting at least one sports club injury out of subjects reporting participation in sports club activities. The injury prevalence in leisure time PA and school-based PA was calculated as a percentage of subjects reporting at least one injury out of all subjects.

### Frequency of physical activity

Physical activity levels were derived from a question ‘How often do you participate in physical activities in your spare time?’ The question was asked separately for five environments: school-based clubs (not physical education classes)/sports clubs/other clubs (e.g. church clubs, scouts)/private sector facilities (e.g. gyms, ski slopes, riding schools, dance schools)/unorganized activities (e.g. backyard games). Five alternatives were provided: ‘less often than once a week or not at all’, ‘once a week, ‘2 to 3 days a week’, ‘4 to 5 days a week’, and ‘6 to 7 days a week’.

### Ethics

The study follows the ethical principles of the Declaration of Helsinki. The online questionnaire was anonymous. Subjects were informed of the aims, methods, voluntary participation, privacy and confidentiality of the collected information. The study protocol has been approved by the Ethics Committee of the University of Jyväskylä. Written informed consent was not required.

### Statistical methods

The data from 2014 and 2016 was combined and used in all the analysis. In the comparison of injury prevalence between years 2014 and 2016, only Finnish-language subjects where analyzed since Swedish-language schools were not represented in the 2014 data. Age groups were used in reporting instead of grade to allow for international comparisons. The 11-year-olds represented all the 5th graders, even if they had already turned 12. Similarly, the 13-year-olds represented the 7th graders and the 15-year-olds represented the 9th graders.

Analyses were performed using SPSS (v.24, SPSS Inc., Chicago, Illinois, USA). The differences in the injury prevalence between the three PA settings were analyzed by Wilcoxon signed rank test. The ordinal logistic regression analysis was used to study the trend of injury prevalence and the associations between age, sex and frequency of PA and injury prevalence. The significance level was set at *p* < 0.05.

## Results

### Injury prevalence in sports clubs, leisure time PA and school-based PA

**Fig. 1:**
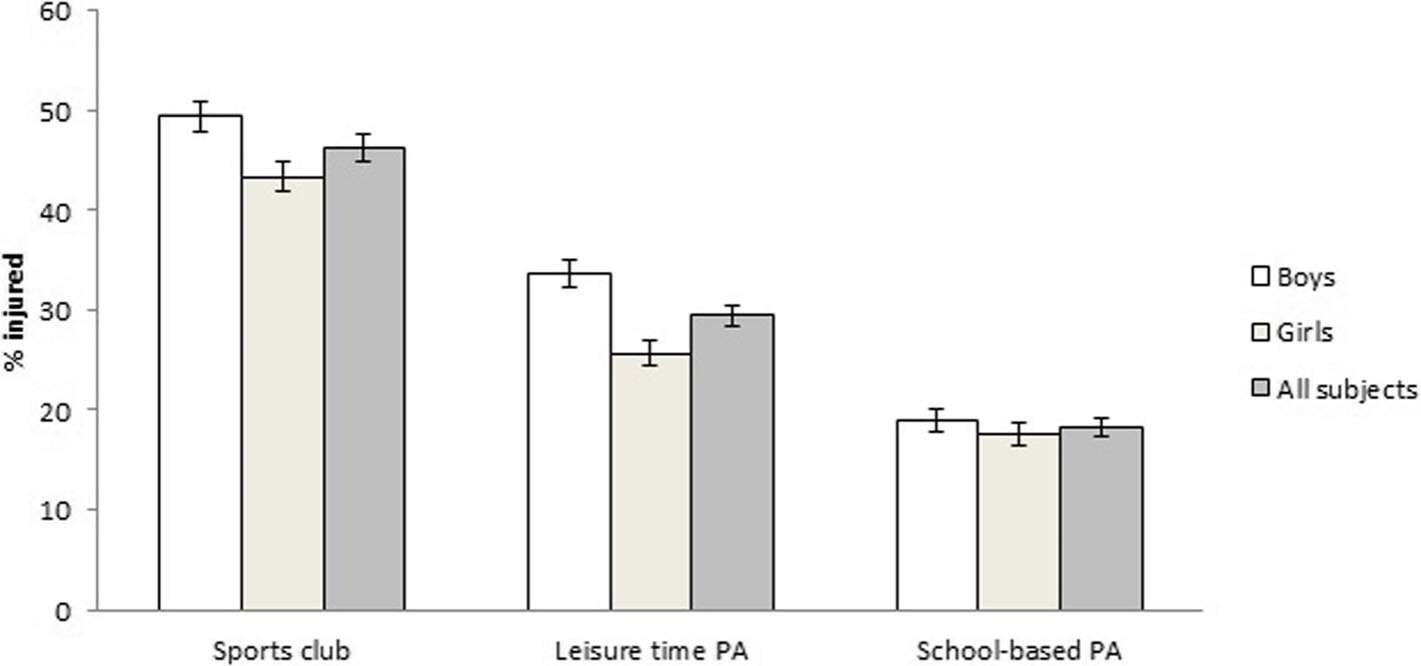
Injury prevalence (%) in the total data in sports club activities, leisure time PA and school-based PA for boys, girls and all subjects presented with 95% confidence interval

In the combined data, 47% (95% CI, 46.2–48.4) of the subjects had suffered at least one PA-related injury during the past 12 months. The injury prevalence was higher in sports club activities (46%, 95% CI 44.8–47.8) than in leisure time PA (30%, 95% CI, 28.5–30.5, *p* < 0.001) and school-based PA (18%, 95% CI, 17.4–19.1, (*p* < 0.001)). In leisure time PA, the injury prevalence was higher than in school-based PA (*p* < 0.001). Injury prevalence in the total data in the three settings is presented in Fig. 1.

### Association between injury prevalence and sex

Boys had significantly more injuries than girls in sports club activities (OR 1.31, 95% CI, 1.17–1.46, *p* < 0.001) and in leisure time PA (OR 1.49, 95% CI, 1.35–1.64, *p* < 0.001). In school-based PA there was no significant difference in injury prevalence between boys and girls. The injury prevalence in different settings among boys and girls is presented in Fig. 1. The odds ratios for injuries in different settings are presented in Table 1.

**Table 1:** The odds ratios (OR) for injuries in different settings for boys and girls

	N	Sports club activities				Leisure time PA				School-based PA			
		Injured (%)	OR	95% CI	*p*	Injured (%)	OR	95% CI	*p*	Injured (%)	OR	95% CI	*p*
Girls	4314	43.4	1			25.7	1			17.6	1		
Boys	4092	49.4	1.31	1.17–1.46	< 0.001	33.7	1.49	1.35–1.64	< 0.001	19.0	1.12	1.00–1.26	0.05

### Association between injury prevalence and age

**Fig. 2:**
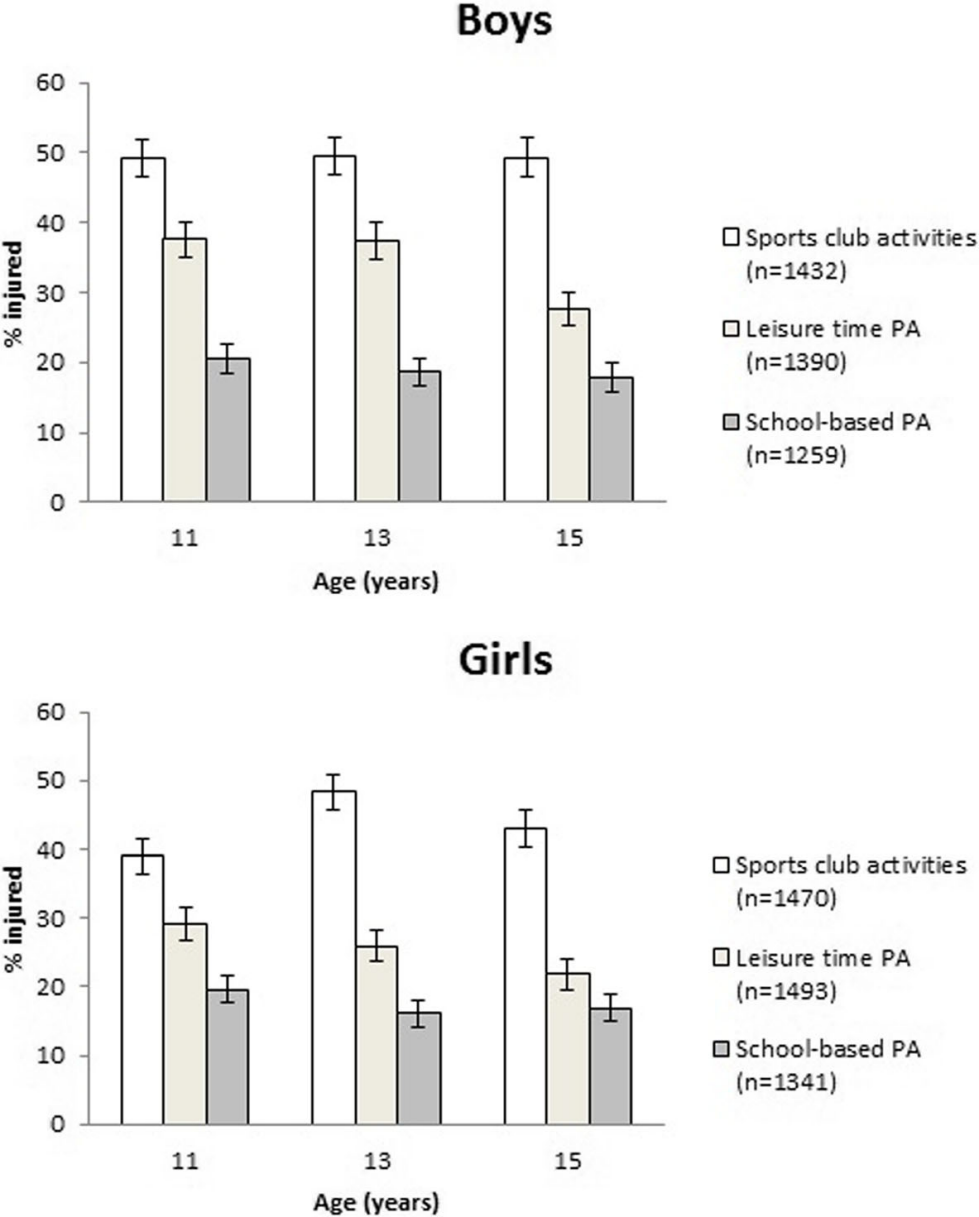
Injury prevalence (%) in sports club activities, leisure time PA and school-based PA by age group for boys and girls presented with 95% confidence interval

Among boys, age was associated with leisure time injuries: the oldest subjects had significantly less injuries compared to the youngest (OR 0.63, 95% CI, 0.53–0.75, *p* < 0.001). Among girls, age was associated with injuries in all three settings. In sports clubs the 13-year-olds had the highest risk of injury (OR 1.48, 96% CI, 1.23–1.77, *p* < 0.001). In leisure time PA the oldest girls had significantly less injuries than the youngest (OR 0.69, 95% CI, 0.59–0.83, *p* < 0.001). In school-based PA the 13-year-olds had less injuries than the 11-year-olds (OR 0.79, 95% CI 0.65–0.97, *p* = 0.02). Injury prevalence in different settings by age groups is presented in Fig. 2.

### Association between injury prevalence and physical activity in sports club and leisure time

In sports club activities and leisure time PA the risk of injury increased significantly along with the frequency of PA. The odds ratios for sports club injuries and leisure time PA injuries in different activity levels are presented in Table 2.

**Table 2:** Age and sex-adjusted odds ratios (OR) and 95% confidence intervals (CI) for injuries in sports club activities and leisure time PA by frequency of activity

Frequency of PA	Sports club activities			Leisure time PA		
	OR	95% CI	*p*	OR	95% CI	*p*
Once a week or less	1			1		
2 to 3 times a week	1.39	1.16–1.66	< 0.001	1.01	0.85–1.20	0.92
4 to 5 times a week	2.29	1.90–2.75	< 0.001	1.40	1.17–1.68	< 0.001
6 to 7 times a week	3.18	2.51–4.01	< 0.001	1.62	1.34–1.96	< 0.001

### The trend of injury prevalence

**Fig. 3:**
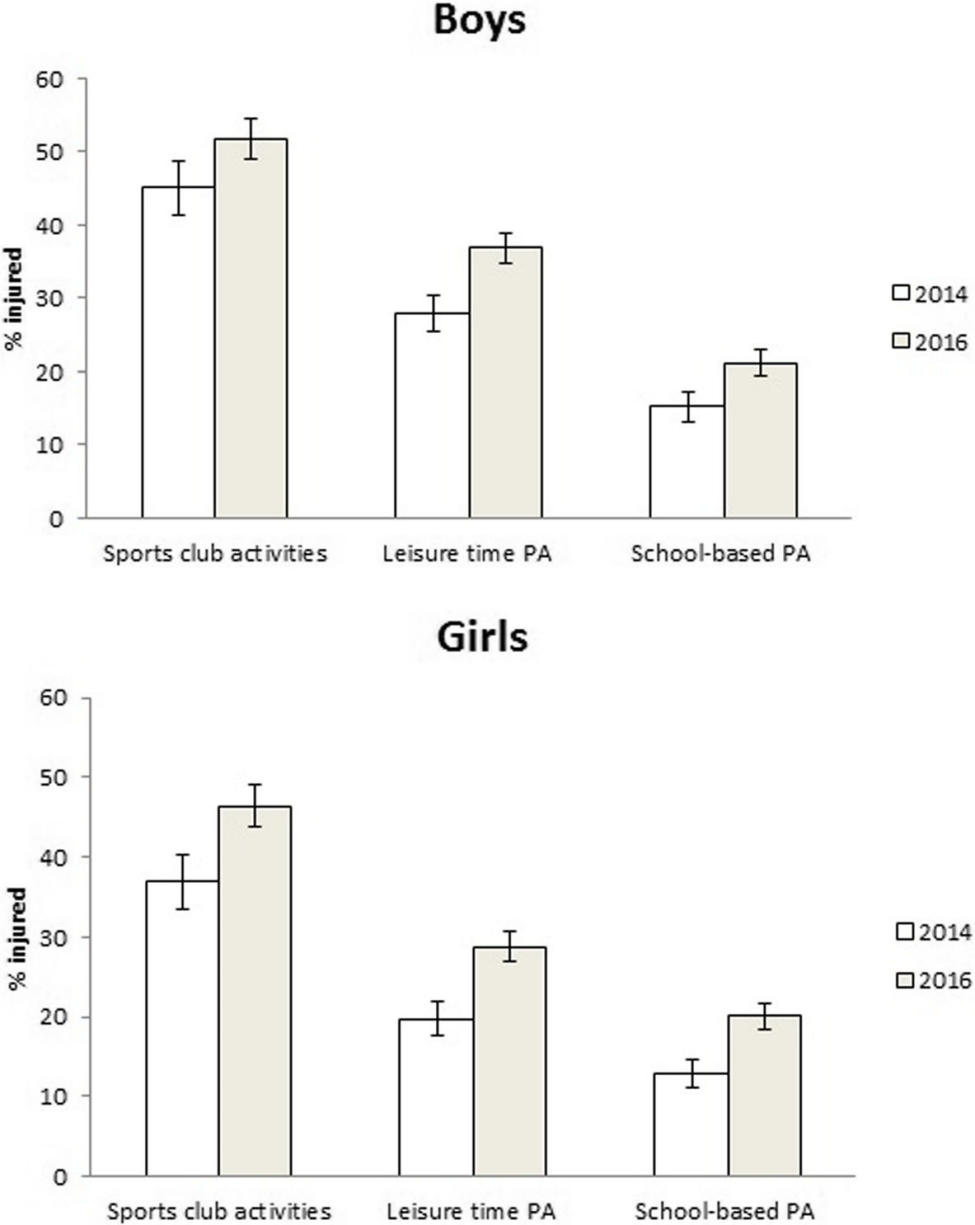
Injury prevalence (%) in sports club activities, leisure time PA and school-based PA in 2014 and 2016 among boys and girls presented with 95% confidence interval

In 2016 the injury prevalence was significantly higher in all three PA settings than in 2014. The prevalence of sports club injuries increased from 41% to 48% (*p* < 0.001). In leisure time PA, the prevalence increased from 24% to 32% (*p* < 0.001). In school-based PA the injury prevalence was 14% in 2014 and 19% in 2016 (*p* < 0.001). Injury prevalence in the three settings 2014 and 2016 for boys and girls is presented in Fig. 3.

## Discussion

This study showed that half of the participating adolescents had been injured in sports club activities, one in three in leisure time PA and one in five in school-based PA during the past 12 months. The most worrisome finding was the increase in injury prevalence in all three PA settings between 2014 and 2016.

Participating in sports club activities seems to be getting more and more popular among adolescents. In the present study, there was a rise from 54% of subject participating in sports club activities in 2014 to 62% in 2016. In the HBSC study in 2010 the rate was 46% [30]. Previous studies on injuries in sports, leisure time PA and school-based PA have consistently reported the highest injury rates in sports out of the three settings [18–20]. In our previous study among adolescents, injury prevalence was highest in sports clubs (28% for boys and 24% for girls), followed by leisure time PA (18% for boys and 13% for girls) and school sports (10% for boys and girls) [20]. Verhagen et al. [19] studied physical activity and sports-related injuries in 10–12-year-old children. They reported the highest injury incidence in sports (0.66 injuries/1000 h of participation), followed by physical education classes (0.50 injuries/1000 h), and leisure time (0.39 injuries/1000 h). Jespersen et al. [18] studied incidence of musculoskeletal injuries in 6–12-year-old children. Incidence was highest in sports (1.57 injuries/1000 h), followed by leisure time PA (0.57 injuries/1000 h) and physical education classes (0.14 injuries/1000 h). The results of this study further support the finding that considerably more injuries occur in sports clubs than in other PA settings. These results, in connection with the growing popularity of sports club participation, highlight the need of more preventative actions in the sports club setting.

To assess the effects of PA promotion on injury prevalence as well as the effectiveness of injury prevention efforts, a monitoring system is needed. Purpose of the biennial LIITU study is to investigate the trend of PA participation and injury prevalence among adolescent Finns. Concerning PA related injuries, these first results are worrisome: in all three setting the injury prevalence was significantly higher in 2016 than in 2014. This finding, while limited to a two-year period, indicate that further efforts are needed to reduce the burden of PA-related injuries to public health and individual wellbeing. However, it must be taken into consideration that the response rate of the National Physical Activity Behaviour Study for Children and Adolescents was lower in 2016 (61%) than 2014 (89%), which could have affected the results in some degree.

Sex was associated with sports club injuries and leisure time PA injuries: boys had significantly more injuries than girls. The association between age and injury risk was influenced by the setting. Among boys we detected an association between age and injury risk only in leisure time PA: the 15-year-old boys had a lower risk of injuries than the 11-year-olds. These results are consistent with those of our previous study [20]. Among girls the 13-year-olds had the highest risk of sports club injury. This is in agreement with the results of Sørensen et al. [31] who reported that among girls the breaking point in sports injury incidence is reached in the age of 13 and both before and after this the incidence was lower. One factor behind the breaking point is presumably the growth spurt [19]. However, this study was unable to demonstrate the breaking point among boys. We didn’t detect any differences in sports club injuries among boys in different age groups while Sørensen reported a breaking point to be reached in the age of 14 [31]. A possible explanation for this might be that Sørensen collected only injuries that required medical treatment in the emergency department and in this study all injuries were collected.

The risk of injuries was associated with the frequency of PA. In sports club activities, we detected a statistically significant increase in the injury risk when the participation in sports club activities was twice a week or more often. In leisure time PA, the risk was higher among those participating four times a week or more often. Adolescents should be encouraged to meet the PA guidelines despite the increased risk of PA-related injuries. Rather PA promotion should also focus on injury prevention [27, 28]. To provide adolescents with the greatest health benefits, the focus should be on the promotion of safe PA. This requires the two separated fields of PA promotion and injury prevention to join forces [5].

These results provide valuable information for public health policy makers. There is a lack of public health policy on prevention of PA-related injuries and the reason for this has been the lack of relevant information [32]. However, now there is research on the negative consequences of PA-injuries [24–26, 33], socioeconomic costs of injuries [34, 35], activities with high injury incidence [36], proven injury prevention methods [11, 37, 38], cost-effectiveness of prevention programs [39], and health-benefits of injury training programs [11]. And there has been progress in injury prevention becoming part of the public health policy: in the US the Centers for Disease Control and Prevention include reducing sports and recreation-related injuries in their National Action Plan for Child Injury Prevention [40]. Our results provide valuable information on the extent of the issue in adolescent population in Finland. In Finland, the Government has appointed increasing PA among school-aged children as one of the key projects in the field of knowledge and education [41]. As such investments are made on the Government level, it is important to monitor the effects these projects have on injury prevalence.

The injury prevalence can be slightly underestimated in this study. The data collection was performed retrospectively and there is always a chance of recall bias. Especially some minor injuries can be difficult to recall and this should be considered a limitation of this work. Another lack of uncertainty is the lack of injury definition in the questionnaire. The subjects could have had different ideas of what constitutes as injury and this could have affected their responses.

## Conclusions

With half of the subjects reporting at least one PA-related injury during the past year, these results indicate that adolescent physical activity-related injuries are a large-scale public health problem. The results show a worrisome rise in injury prevalence in recent years. From a public health standpoint, there is an urgent need to invest in injury prevention to reverse this trend. With sport club activities gaining popularity and nearly half of the adolescents participating in sports club activities getting injured, the greatest effort should be directed there. Preventative measures are necessary among boys and girls equally and in all age groups.

## Abbreviations

HBSC: Health Behaviours in School-Aged Children study
PA: Physical activity
